# Performance Comparison of Object Detection Networks for Shrapnel Identification in Ultrasound Images

**DOI:** 10.3390/bioengineering10070807

**Published:** 2023-07-05

**Authors:** Sofia I. Hernandez-Torres, Ryan P. Hennessey, Eric J. Snider

**Affiliations:** U.S. Army Institute of Surgical Research, JBSA Fort Sam Houston, San Antonio, TX 78234, USA

**Keywords:** ultrasound imaging, image interpretation, artificial intelligence, machine learning, deep learning, object detection, shrapnel, neurovascular

## Abstract

Ultrasound imaging is a critical tool for triaging and diagnosing subjects but only if images can be properly interpreted. Unfortunately, in remote or military medicine situations, the expertise to interpret images can be lacking. Machine-learning image interpretation models that are explainable to the end user and deployable in real time with ultrasound equipment have the potential to solve this problem. We have previously shown how a YOLOv3 (You Only Look Once) object detection algorithm can be used for tracking shrapnel, artery, vein, and nerve fiber bundle features in a tissue phantom. However, real-time implementation of an object detection model requires optimizing model inference time. Here, we compare the performance of five different object detection deep-learning models with varying architectures and trainable parameters to determine which model is most suitable for this shrapnel-tracking ultrasound image application. We used a dataset of more than 16,000 ultrasound images from gelatin tissue phantoms containing artery, vein, nerve fiber, and shrapnel features for training and evaluating each model. Every object detection model surpassed 0.85 mean average precision except for the detection transformer model. Overall, the YOLOv7tiny model had the higher mean average precision and quickest inference time, making it the obvious model choice for this ultrasound imaging application. Other object detection models were overfitting the data as was determined by lower testing performance compared with higher training performance. In summary, the YOLOv7tiny object detection model had the best mean average precision and inference time and was selected as optimal for this application. Next steps will implement this object detection algorithm for real-time applications, an important next step in translating AI models for emergency and military medicine.

## 1. Introduction

Medical imaging is an essential tool for diagnosing diseases such as COVID-19 [1,2], cancer [3,4], and triaging the severity of a subject’s condition in emergency medicine [5,6]. At the point of injury and in austere or remote environments, oftentimes only ultrasound (US) imaging is available due to its small size and minimal power consumption [6]. US image acquisition can seem to be simple, but capturing the correct image view requires extensive experience. This also applies to image interpretation, as skilled radiographers often must interpret and make triage or diagnostic decisions. In remote medical scenarios, such as combat casualty care, medical expertise to interpret images is often lacking, resulting in US imaging not getting deployed to the frontlines and, thus, not being able to assist with making critical triage decisions about a subject’s condition [7].

In response, machine-learning (ML) algorithms have been proposed to automate image interpretation for US images [8,9]. Methods have been used to automate COVID-19 classification from an US scan of the chest [10] and identify free fluid in the abdomen [11], and we have shown how shrapnel can be identified in phantom or swine tissue [12]. These examples provide evidence of the possibility for ML to simplify US image interpretation for the end user, but there are additional criteria for remote US imaging applications as end users may not have sufficient training. First, the ML model’s output needs to be easily understandable to the end user and explainable to the rear echelon medical providers. Simple classification models where no indication is provided as to what in the US image indicated a certain diagnosis will likely be met with resistance. Instead, ML object detection models are more suitable for this task since a bounding box is provided as the region of interest to assist with the diagnostic decision. Second, the ML image interpretation model needs to be integrated into US equipment so that real-time object detection tracking is possible regardless of data connectivity. This will require lighter ML models needing reduced computing capabilities for quicker image interpretation while still maintaining high accuracy. As a result, predicted bounding boxes can be overlaid on US images in real time for assisted diagnosis.

We have previously shown how a YOLOv3 object detection neural network ML models can accurately identify shrapnel, artery, vein, and nerve fiber anatomical features in a tissue phantom model. Here, we compare the performance of YOLOv3 against other object detection approaches to identify which model optimally performs in terms of inference time (prediction speed) and overall precision.

### 1.1. Overview of Object Detection for Ultrasound Imaging

Multiple review papers have evaluated current state-of-the-art object detection ML models in general [13,14,15] as well as for medical applications [16,17]. Specifically for ultrasound imaging, object detection has been used for a wide range of applications. This includes tumor identification in tissue [18,19], obstetric medicine [20], and musculoskeletal applications [21]. For vessel identification applications, Brattain et al., utilized a YOLOv3tiny network for identifying arteries and veins in phantom and swine tissue [22]. The YOLOtiny networks are optimized for real-time performance allowing for guidance of vascular access procedures to occur in near real time after integration with ultrasound hardware. Smistad et al. developed an AlexNet-based object detection model to identify elliptical vessel overlays on arteries and veins with vessel detection in 46 ms inference time [23]. Similarly, Zeng et al. implemented an object detection model in OpenCV capable of identifying vessels in 12 ms for venipuncture applications [24]. These prior studies provide evidence that object detection models, when properly selected and tuned, can be suitable for ultrasound applications.

### 1.2. Object Detection Architectures

The YOLO (You Only Look Once) series of object detection models use deep convolutional neural networks to learn features that are then used to identify objects in images [25]. YOLO utilizes a single neural network for classification and bounding box predictions that improves on the speed of R-CNN and other conventional object detection models [26]. We recently used YOLOv3 for shrapnel detection in tissue phantom images as YOLOv3 improves on the performance of the initial YOLO model by using a Darknet classifier backbone [27]. Building on this previous work, we will re-evaluate YOLOv3 to determine how it compares against YOLOv7 and YOLOv7tiny. YOLOv7 is the most recent iteration of the YOLO series and is the state of the art in object detection, outperforming most real-time object detectors [28]. YOLOv7tiny is a modified version of this network oriented toward edge computing (i.e., real-time detection) with only a fraction of the parameters yet still maintaining comparable performance [28]. Fewer parameters reduce the chance of overfitting in simpler imaging applications and have improved performance metrics such as mean average precision (mAP) when dealing with small objects in ultrasound images [20]. The improvement in performance compared with other object detectors has made YOLOv7 and YOLOv7-tiny popular for medical imaging applications for chest abnormalities [29], kidney diseases [30], and fetal cardiac objects [20].

Another recent class of ML models has been focused on integrating a self-attention mechanism in model architecture [31]. Self-attention is a technique trying to mimic humans focus and gives greater weight to keywords in sentences or regions of image when processing information that is often missing in traditional ML models. These self-attention models, termed transformers, were initially used for improving natural language processing problems [32], but they have more recently been used in the image processing tasks, termed vision transformers (ViTs) [33,34]. Instead of separately looking at each pixel of an image, images are split into fixed-size patches so that image context can be preserved. Transformer encoder and decoder capabilities can be merged into traditional CNN architectures to allow for initial feature identification followed by the self-attention mechanisms of the transformer features [35,36]. ViTs have more widely been used for image segmentation and classification tasks, but Carion et al. highlighted a DEtection TRansformer (DETR) framework [37] that has been used for medical image segmentation applications [38].

A different widely used object detection architecture is EfficientDet, a weighted bidirectional feature network with a customized scaling method [39,40]. EfficientDet outperformed YOLOv3, but other studies have shown that fine-tuning and optimizing model training for an EfficientDet architecture is resource intensive [41]. This object detection model is based on EfficientNet, which can vary in size from EfficientNet-B0 at 5.3 million parameters to EfficientNet-B7 at 66 million parameters [40]. Once trained and deployed, EfficientDet model accuracies and inference times vary based on application and selected model version. For instance, EfficientDet-D0 has 2.5 billion FLOPs (floating point operations per second) and COCO mAP of 0.33, while EfficientDet-D7 was much slower at 325 billion FLOPs and an improved COCO mAP of 0.52 [39]. For this ultrasound imaging application, EfficientDet-D2 was selected for its balanced trade-off between speed and performance.

A summary of the features for the selected models as well as the rationale for their selection is highlighted in Table 1. We selected 5 models to compare for this application: YOLOv3, YOLOv7, YOLOv7tiny, DETR-R50, and EfficientDet-D2.

## 2. Materials and Methods

### 2.1. Dataset Prep

Ultrasound images were obtained from a previously developed ultrasound tissue phantom dataset [42]. Briefly, all imaging was performed underwater with the HL50x (Sonosite, Fujifilm, Bothell, WA, USA) probe with the Sonosite Edge ultrasound system (Sonosite, Fujifilm, Bothell, WA, USA). Tissue phantoms were composed of 10% gelatin in a 2:1 evaporated milk-to-water ratio made in two layers: an internal layer containing 0.25% flour concentration and agarose fragments in the bulk for increased heterogeneity in the imaging dataset, and an external layer with 0.10% flour concentration. After the gelatin solidified, vessel channels were made using a biopsy punch in the shape of a vein, artery, or nerve fiber. For the nerve fiber bundle, the channel was filled with 0.5% flour concentration of the bulk gelatin solution. The shrapnel object was inserted under water using surgical forceps when creating the shrapnel image dataset. Each individual phantom was considered a different subject, and images from a total of 6 subjects were pooled for this study. Of these 6 phantoms, half were a simple phantom with or without shrapnel present during imaging, and the rest were a complex phantom containing a neurovascular bundle (vein, artery, and nerve fiber) with or without shrapnel. A total of approximately 16,000 images were used across the 6 subjects.

**Figure 1:**
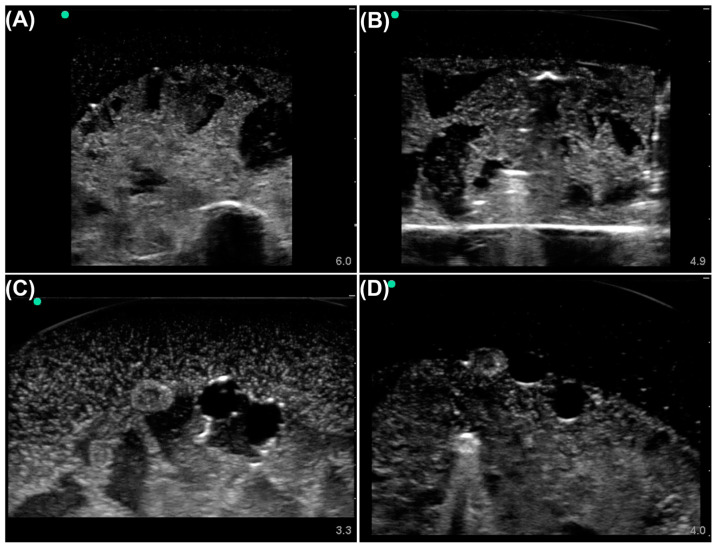
Representative phantom images from each labeling category. Tissue phantom ultrasound images were divided into four categories: (**A**) Baseline simple phantom, no shrapnel or neurovascular features. (**B**) Shrapnel in the simple phantom, without neurovascular features. (**C**) Baseline complex phantom with vein, artery, and nerve objects. (**D**) Shrapnel in the complex phantom with vein, artery, and nerve objects. Representative US pictures were increased in brightness for ease of image interpretation.

Images were then divided into four categories—(i) no shrapnel or neurovascular features, (ii) shrapnel and no neurovascular features, (iii) no shrapnel with neurovascular features, and (iv) shrapnel and neurovascular features (Figure 1). Ground-truth bounding boxes were generated with the Image Processing Toolbox in MATLAB (Mathworks, R2021b, Natick, MA, USA). The labeling was split into two sessions, one for shrapnel only (category ii) and one for vein, artery, nerve, and shrapnel for images from the complex phantom (categories iii and iv). The ground-truth label arrays from both sessions were then exported from MATLAB, merged, and converted to a CSV file. The ground-truth bounding boxes were adapted to the YOLOv3 labeling format, creating individual text files of the respective ground-truth bounding box(es) for each image, used in YOLOv3 training. For all other object detection models, the ground-truth labels were converted from YOLOv3 formatting to the COCO (or JSON) format.

### 2.2. Object Detection Model Preparation and Training

All object detection models chosen were imported with pretrained MS COCO weights for trainable parameters. Images for training were kept at the original size of the dataset—512 × 512—for training all models. The image dataset was split using an 80:10:10 ratio for training, validation, and testing, respectively. The split of images for training went through a fine-tuning phase in which several augmentations were applied. The augmentations included flips (rotation about the y-axis), cropping, HSV (hue saturation value) distortion, and shifting. The magnitude for each augmentation reflected the original training parameters of the pretrained models. Other training parameters (e.g., validation frequency, learning rate, and solver) followed the established pretrained model’s architecture with one exception: the training batch size for DETR was reduced to 8 images due to computational restrictions. Model fine-tuning for the shrapnel dataset used images from the training and validation split and was allowed to continue for a maximum of 300 epochs with early stopping based on failure to improve on validation loss for 5 consecutive epochs.

### 2.3. Backend Performance Evaluation and Real-Time Testing

**Figure 2:**
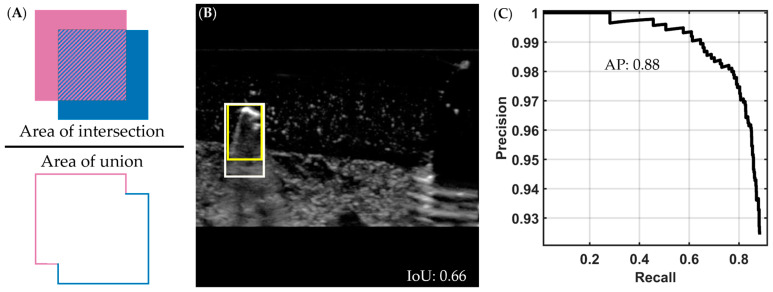
Visual description of intersection over union and average precision metrics. (**A**) Intersection over union (IoU) is calculated by dividing the intersection (pattern region in the numerator) of the ground-truth and predicted bounding boxes by the union of the two (area of both boxes combined, shown in denominator). (**B**) Representative ultrasound image showing a predicted (darker yellow) and ground-truth (lighter yellow) bounding box around a shrapnel object, with an IoU score of 0.66. The representative US image was increased in brightness for ease of interpretation. (**C**) A precision-recall curve from which we obtain the average precision (AP) by calculating area under the curve; in the example shown, the AP is 0.88. To generate this curve, precision is calculated as the total of true positives divided by all the positive predictions and recall as the total of true positives divided by the true positives and false negatives, for multiple classifier thresholds.

Once the models were trained, their performance was compared by using the blind testing images from the shrapnel dataset. The key performance metrics for comparing model performance were mean average precision (mAP), intersection over union (IoU), and inference time (in milliseconds). IoU is the ratio of the intersection between the ground-truth box and predicted object box to the total area, or union, between the ground-truth box and predicted object box (Figure 2A,B). A higher value of IoU, i.e., the closer it is to 1, indicates better agreement between the prediction and ground-truth bounding box. Average precision is the area under the precision–recall curve, which is constructed using different confidence values for a single class (Figure 2C). The mAP value is calculated across all classes for the object detector (vein, artery, nerve, and shrapnel in our case) [43]. IoU and mAP can be combined, so that different IoU values or ranges can be used as thresholds during mAP calculation; for example, mAP at an IoU of 0.5 (mAP@0.50) will deem every image with an IoU threshold below 0.5 as a false positive and calculate the mAP with the new true positive total.

Inference times—defined as the time to make a prediction per image—were determined across the entire test dataset for each model using the same computer hardware setup. A NVIDIA GeForce RTX 3090 Ti 24Gb VRAM system with Intel i9-12900k and 64 GB RAM with Ubuntu 22.04 operating system was used for determining the inference time.

## 3. Results

Testing results were tabulated for mAP (see Section 2.3, Figure 2) at various IoU scores (Table 2). Overall, all models were able to correctly identify the vein, artery, nerve, or shrapnel objects in the ultrasound images with a greater than 0.40 mAP@0.50:0.95. YOLOv7tiny had the highest score at 0.615 mAP@0.50:0.95. However, all the models failed to consistently identify objects when a higher IoU threshold of 0.95 was applied to mAP. With a higher IoU threshold, the subjectivity in precisely determining the ground-truth bounding box labels greatly impacted the performance. Using a 0.50 IoU threshold for mAP, YOLOv7tiny outperformed the other models with a 0.957 mAP@0.50, while DETR was the worst performing model for this application, with a mAP@0.50 of 0.643.

Comparing model inference times (Table 2), DETR and EfficientDet-D2 took 34.13 and 22.7 ms/image, respectively, compared with 7.87 ms on average for the YOLO family of models. YOLOv7tiny performed the best with an inference time of 5.68 ms/image. This large disparity in inference time makes EffecientDet-D2 and DETR less suitable for this application’s future transition to real-time integration with US hardware.

To further compare performance of the various models and why some models fared better for this application, performance metrics were captured for training images in addition to test image datasets (Table 3). As expected, all models performed better with training images. The testing-to-training ratio for each mAP score highlights the performance gap between the two image datasets as an indicator of model overfitting. DETR had the lowest ratio, indicating that it has the greatest accuracy drop between blind test images and training images. Conversely, YOLOv7tiny was closest to one for this ratio at both mAP metrics.

**Figure 3:**
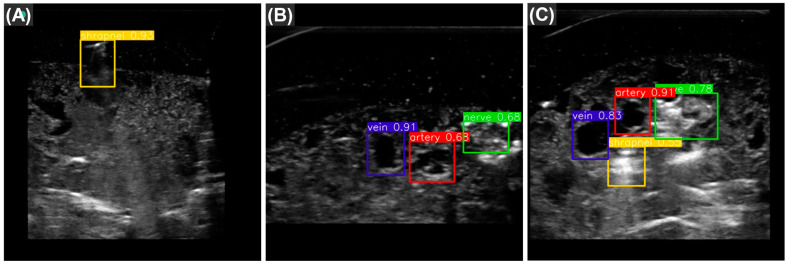
Representative test predictions using YOLOv7tiny. The three images are example object detection predictions for shrapnel (yellow), vein (blue), artery (red), and nerve fiber (green) taken from YOLOv7tiny. Images are shown for each of three categories: (**A**) shrapnel in simple phantom, with neurovascular features; (**B**) baseline complex phantom, with vein artery and nerve; and (**C**) shrapnel in complex phantom. The numbers adjacent to the predictions represent the confidence measure of that prediction.

Overall, as YOLOv7tiny had the highest mAP scores and quickest inference time, it was selected as the most optimal for this ultrasound imaging application. Representative predictions for each category of ultrasound image are shown with bounding box predictions using YOLOv7tiny (Figure 3). In addition, performance metrics for each label category were calculated (Table 4). The model most struggled with identifying shrapnel as it had much lower performance metrics compared with vein, artery, and nerve fiber.

## 4. Discussion

Ultrasound imaging can be a critical tool for triage and diagnosis at or near the point of injury if the skill threshold for image interpretation can be lowered. Image classification algorithms have been extensively used for this purpose, but they struggle from an explainable AI perspective as the prediction is normally provided with no rationale. Instead, we focus on object detection models, as the bounding box highlights the features in the image being tracked. We compared different model architectures for shrapnel tracking in ultrasound images to identify which generates the most suitable model in terms of precision and speed.

Overall, the different models performed in line with their MS COCO dataset performance with a few exceptions. For instance, YOLOv7tiny outperformed all other models on mAP@0.50, mAP@0.50:0.95, and inference time metrics. This may be considered unexpected as YOLOv7tiny is a smaller neural network with fewer trainable parameters than the other models we evaluated. However, in our case, the other models may, in part, be overfitting the small and relatively simple training dataset given the size of these networks. Overfitting by DETR and other models was shown by how pronounced the drop in the mAP metric was for the training versus test data, whereas YOLOv7tiny showed a more subtle decrease in the same metric. Training and testing results should agree, and an imbalance between them supports overtraining. This can result in the model fitting the noise in the data as opposed to generalizing across this variability, leading to poor performance in blind datasets. In the future, this issue can be reduced by using different early stopping criteria or by introducing a more complex dataset, e.g., more identifiable structures or additional subject variability for the object detection models to predict on.

The primary goal for this study was to identify an object detection model suitable for real-time application both in terms of speed and precision. Given this, YOLOv7tiny will be selected for future advances, such as implementation onto a single board computer for the sake of portability. The model has less trainable parameters than the other models evaluated, but this likely helped prevent overfitting in this relatively simple image interpretation application. The model generated with YOLOv7tiny outperformed all others in every evaluation criterion. However, given its relatively high performance on precision, a smaller iteration of EfficientDet may also be considered to overcome its performance as the model with the slowest inference time. For example, the baseline EfficientDet-D0 still outperforms versions such as EfficientDet-D1 and EfficientDetD2 in other ultrasound applications in terms of accuracy [44]. Our study supports EfficientDet-D2 as a high accuracy model implying that a different version of EfficientDet with less trainable parameters can improve its inference time and is worth considering for future work.

While we selected a model suitable for future work, our study had some limitations. First, the image sets were collected in an US tissue phantom instead of animal or human tissue. The tissue phantom has heterogeneous complexities in the tissue bulk making it a challenging imaging platform to detect shrapnel, but it lacks the tissue level organization seen in biological tissue. While tissue phantoms can be important first steps in developing medical image analysis applications such as this, the lack of biological complexity may require transfer-learning the model prior to use in animal or human tissue. However, we have previously been able to train an AI model to recognize shrapnel in swine tissue by supplementing phantom training image sets with only 10% swine images [42]. Second, the testing data for this study did not contain entire subject holdouts, which may reduce the model’s performance with new subjects. This will be critical to evaluate prior to real-time implementation, as subject variability is clinically important, and a deployable model must be generalized to handle this variability. Additional data augmentation or ensemble prediction approaches may be needed to make objection models more generalized going forward [45]. Third, a limited number of object detection models were evaluated in this study, and as a result, a more optimal model may exist. The goal here was to evaluate a wide range of model architectures, but having identified YOLOv7tiny as the most optimal, additional comparison of similar model setups or hyperparameter optimization can result in further improvements.

Next steps for this study will take three primary directions. First, object detection models will be implemented for use in a real-time format. This will utilize single board computers and a video stream from ultrasound equipment, resulting in real-time bounding box overlay for easy image interpretation. Second, models will be trained on swine shrapnel images, furthering the translation and animal tissue validation of the object detection model. Lastly, object detection models for ultrasound image interpretation can be extended into others at or near the point of injury for triage or diagnostic applications, such as the extended focused assessment with sonography in trauma (eFAST) examination for identifying free fluid or air in the chest and the abdomen.

## 5. Conclusions

In summary, various object detection AI models were able to track neurovascular features and shrapnel with variable success. YOLOv7tiny had the best accuracy and quickest inference time, indicating a clear object detection model for this imaging application. Combining this model with additional training on human and animal data sets as well as integration into a real-time application will allow for ultrasound image interpretation without the need for a skilled radiologist. This can enable ultrasound imaging to be more regularly used at or near the point of injury to triage and diagnose patient status, critical for remote or military medicine. For instance, object detection can be used to evaluate the proximity of shrapnel to vital neurovascular features to provide granularity in triage decision when resources are limited. While this level of resource management may be less pertinent in most first world environments, it can mean the difference between life and death on the battlefield or in remote medical situations.

## Figures and Tables

**Table 1:** Summary of object detection architectures used in this study.

Model	Backbone Architecture	Parameters	COCO mAP@50
YOLOv3	Darknet-53	65.2 M	57.9
YOLOv7	E-ELAN (Extended Efficient Layer Aggregation Network) computational blocks	36.9 M	69.7
YOLOv7tiny	E-ELAN computational blocks	6.2 M	56.7
DETR-R50	ResNet-50	41 M	42.04
EfficientDet-D2	EfficientNet-B2	8.1 M	62.7

**Table 2:** Performance metrics for all trained models. Summary of results for test images showing mean average precision scores at different intersection-over-union thresholds as well as inference times for each trained model.

Model	mAP@0.50	mAP@0.95	mAP@0.50:0.95	Inference Time (ms/Image)
YOLOv3	0.889	0.00013	0.490	9.72
YOLOv7	0.921	0.00189	0.567	8.22
YOLOv7tiny	0.957	0.00211	0.615	5.68
DETR	0.643	0.003	0.423	34.13
EfficientDet-D2	0.909	0.003	0.562	22.74

**Table 3:** Performance metrics for training images for all object detection models evaluated. Summary of results showing mean average precision at different intersection-over-union thresholds and ratios between these scores obtained with the testing dataset to the training dataset.

Model	mAP@0.50		mAP@0.50:0.95	
	Training Result	Testing to Training Ratio	Training Result	Testing to Training Ratio
YOLOv3	0.978	0.909	0.581	0.843
YOLOv7	0.994	0.927	0.733	0.774
YOLOv7tiny	0.990	0.967	0.721	0.853
DETR	0.742	0.867	0.627	0.675
EfficientDet-D2	0.988	0.920	0.725	0.775

**Table 4:** Training performance metrics by category for YOLOv7tiny. Total number of images for each category is shown along with recall and precision scores. Average precision is calculated with a 0.50 IoU threshold for each category.

Category	Count	Recall	Precision	AP:0.50
Vein	1080	0.955	0.963	0.981
Artery	1115	0.981	0.985	0.993
Nerve	1113	0.954	0.949	0.937
Shrapnel	1378	0.819	0.819	0.858

## Data Availability

The datasets generated during and/or analyzed during the current study are available from the corresponding author upon reasonable request.

## References

[1] Gil-Rodríguez J., Pérez de Rojas J., Aranda-Laserna P., Benavente-Fernández A., Martos-Ruiz M., Peregrina-Rivas J.-A., Guirao-Arrabal E. (2022). Ultrasound Findings of Lung Ultrasonography in COVID-19: A Systematic Review. Eur. J. Radiol..

[2] European Society of Radiology (ESR) (2021). The Role of Lung Ultrasound in COVID-19 Disease. Insights Imaging.

[3] Wang X., Yang M. (2021). The Application of Ultrasound Image in Cancer Diagnosis. J. Healthc. Eng..

[4] Zhang G., Ye H.-R., Sun Y., Guo Z.-Z. (2022). Ultrasound Molecular Imaging and Its Applications in Cancer Diagnosis and Therapy. ACS Sens..

[5] Marin J., Abo A., Doniger S., Fischer J., Kessler D., Levy J., Noble V., Sivitz A., Tsung J., Vieira R. (2015). Point-of-care ultrasonography by pediatric emergency physicians. Ann. Emerg. Med..

[6] (1990). American College of Emergency Physicians Council Resolution on Ultrasound. ACEP News.

[7] Townsend S., Lasher W. (2018). The U.S. Army in Multi-Domain Operations 2028.

[8] Micucci M., Iula A. (2022). Recent Advances in Machine Learning Applied to Ultrasound Imaging. Electronics.

[9] Liu S., Wang Y., Yang X., Lei B., Liu L., Li S.X., Ni D., Wang T. (2019). Deep Learning in Medical Ultrasound Analysis: A Review. Engineering.

[10] Diaz-Escobar J., Ordóñez-Guillén N.E., Villarreal-Reyes S., Galaviz-Mosqueda A., Kober V., Rivera-Rodriguez R., Rizk J.E.L. (2021). Deep-Learning Based Detection of COVID-19 Using Lung Ultrasound Imagery. PLoS ONE.

[11] Lin Z., Li Z., Cao P., Lin Y., Liang F., He J., Huang L. (2022). Deep Learning for Emergency Ascites Diagnosis Using Ultrasonography Images. J. Appl. Clin. Med. Phys..

[12] Snider E.J., Hernandez-Torres S.I., Boice E.N. (2022). An Image Classification Deep-Learning Algorithm for Shrapnel Detection from Ultrasound Images. Sci. Rep..

[13] Zaidi S.S.A., Ansari M.S., Aslam A., Kanwal N., Asghar M., Lee B. (2022). A Survey of Modern Deep Learning Based Object Detection Models. Digit. Signal Process..

[14] Zhao Z.-Q., Zheng P., Xu S., Wu X. (2019). Object Detection with Deep Learning: A Review. IEEE Trans. Neural Netw. Learn. Syst..

[15] Wu X., Sahoo D., Hoi S.C. (2020). Recent Advances in Deep Learning for Object Detection. Neurocomputing.

[16] Kaur A., Singh Y., Neeru N., Kaur L., Singh A. (2021). A Survey on Deep Learning Approaches to Medical Images and a Systematic Look up into Real-Time Object Detection. Arch. Comput. Methods Eng..

[17] Latif J., Xiao C., Imran A., Tu S. (2019). Medical Imaging Using Machine Learning and Deep Learning Algorithms: A Review. Proceedings of the 2019 2nd International Conference on Computing, Mathematics and Engineering Technologies (iCoMET).

[18] Cao Z., Duan L., Yang G., Yue T., Chen Q., Fu H., Xu Y., Wu G., Munsell B.C., Zhan Y., Bai W., Sanroma G., Coupé P. (2017). Breast Tumor Detection in Ultrasound Images Using Deep Learning. Patch-Based Techniques in Medical Imaging.

[19] Chiang T.-C., Huang Y.-S., Chen R.-T., Huang C.-S., Chang R.-F. (2019). Tumor Detection in Automated Breast Ultrasound Using 3-D CNN and Prioritized Candidate Aggregation. IEEE Trans. Med. Imaging.

[20] Iriani Sapitri A., Nurmaini S., Naufal Rachmatullah M., Tutuko B., Darmawahyuni A., Firdaus F., Rini D.P., Islami A. (2023). Deep Learning-Based Real Time Detection for Cardiac Objects with Fetal Ultrasound Video. Inform. Med. Unlocked.

[21] Tang Y., Chen H., Qian L., Ge S., Zhang M., Zheng R. Detection of Spine Curve and Vertebral Level on Ultrasound Images Using DETR. Proceedings of the 2022 IEEE International Ultrasonics Symposium (IUS).

[22] Brattain L.J., Pierce T.T., Gjesteby L.A., Johnson M.R., DeLosa N.D., Werblin J.S., Gupta J.F., Ozturk A., Wang X., Li Q. (2021). AI-Enabled, Ultrasound-Guided Handheld Robotic Device for Femoral Vascular Access. Biosensors.

[23] Smistad E., Løvstakken L. (2016). Vessel Detection in Ultrasound Images Using Deep Convolutional Neural Networks. Proceedings of the Deep Learning and Data Labeling for Medical Applications: First International Workshop, LABELS 2016, and Second International Workshop, DLMIA 2016, Held in Conjunction with MICCAI 2016.

[24] Zeng Y., Wang H., Sha M., Lin G., Long Y., Liu Y. Object Detection Algorithm of Vein Vessels in B-Mode Ultrasound Images. Proceedings of the 2022 7th International Conference on Control and Robotics Engineering (ICCRE).

[25] Jiang P., Ergu D., Liu F., Cai Y., Ma B. (2022). A Review of Yolo Algorithm Developments. Procedia Comput. Sci..

[26] Redmon J., Divvala S., Girshick R., Farhadi A. You Only Look Once: Unified, Real-Time Object Detection. https://arxiv.org/abs/1506.02640v5.

[27] Redmon J., Farhadi A. (2018). YOLOv3: An Incremental Improvement. arXiv.

[28] Wang C.-Y., Bochkovskiy A., Liao H.-Y.M. YOLOv7: Trainable Bag-of-Freebies Sets New State-of-the-Art for Real-Time Object Detectors. https://arxiv.org/abs/2207.02696v1.

[29] Sun K.X., Cong C. Research On Chest Abnormality Detection Based On Improved YOLOv7 Algorithm. Proceedings of the 2022 IEEE International Conference on Bioinformatics and Biomedicine (BIBM).

[30] Bayram A.F., Gurkan C., Budak A., Karataş H. (2022). A Detection and Prediction Model Based on Deep Learning Assisted by Explainable Artificial Intelligence for Kidney Diseases. EJOSAT.

[31] Vaswani A., Shazeer N., Parmar N., Uszkoreit J., Jones L., Gomez A.N., Kaiser Ł., Polosukhin I. (2017). Attention Is All You Need. Adv. Neural Inf. Process. Syst..

[32] Yang B., Wang L., Wong D.F., Shi S., Tu Z. (2021). Context-Aware Self-Attention Networks for Natural Language Processing. Neurocomputing.

[33] Park N., Kim S. (2022). How Do Vision Transformers Work?. arXiv.

[34] Zhou D., Kang B., Jin X., Yang L., Lian X., Jiang Z., Hou Q., Feng J. (2021). Deepvit: Towards Deeper Vision Transformer. arXiv.

[35] Wu H., Xiao B., Codella N., Liu M., Dai X., Yuan L., Zhang L. Cvt: Introducing Convolutions to Vision Transformers. Proceedings of the IEEE/CVF International Conference on Computer Vision.

[36] Yuan K., Guo S., Liu Z., Zhou A., Yu F., Wu W. Incorporating Convolution Designs into Visual Transformers. Proceedings of the IEEE/CVF International Conference on Computer Vision.

[37] Carion N., Massa F., Synnaeve G., Usunier N., Kirillov A., Zagoruyko S. (2020). End-to-End Object Detection with Transformers. Proceedings of the Computer Vision–ECCV 2020: 16th European Conference.

[38] Hatamizadeh A., Tang Y., Nath V., Yang D., Myronenko A., Landman B., Roth H.R., Xu D. Unetr: Transformers for 3d Medical Image Segmentation. Proceedings of the IEEE/CVF Winter Conference on Applications of Computer Vision.

[39] Tan M., Pang R., Le Q.V. EfficientDet: Scalable and Efficient Object Detection. Proceedings of the IEEE/CVF Conference on Computer Vision and Pattern Recognition.

[40] Tan M., Le Q.V. (2020). EfficientNet: Rethinking Model Scaling for Convolutional Neural Networks. arXiv.

[41] Du R., Chen Y., Li T., Shi L., Fei Z., Li Y. (2022). Discrimination of Breast Cancer Based on Ultrasound Images and Convolutional Neural Network. J. Oncol..

[42] Hernandez-Torres S.I., Boice E.N., Snider E.J. (2022). Using an Ultrasound Tissue Phantom Model for Hybrid Training of Deep Learning Models for Shrapnel Detection. J. Imaging.

[43] Padilla R., Netto S.L., da Silva E.A.B. A Survey on Performance Metrics for Object-Detection Algorithms. Proceedings of the 2020 International Conference on Systems, Signals and Image Processing (IWSSIP).

[44] Medak D., Posilović L., Subašić M., Budimir M., Lončarić S. (2021). Automated Defect Detection From Ultrasonic Images Using Deep Learning. IEEE Trans. Ultrason. Ferroelectr. Freq. Control.

[45] Snider E.J., Hernandez-Torres S.I., Hennessey R. (2023). Using Ultrasound Image Augmentation and Ensemble Predictions to Prevent Machine-Learning Model Overfitting. Diagnostics.

